# Splenium of Corpus Callosum: Patterns of Interhemispheric Interaction in Children and Adults

**DOI:** 10.1155/2013/639430

**Published:** 2013-03-13

**Authors:** Maria G. Knyazeva

**Affiliations:** ^1^LREN, Department of Clinical Neuroscience, Centre Hospitalier Universitaire Vaudois (CHUV) and University of Lausanne, CH-1011 Lausanne, Switzerland; ^2^Department of Radiology, Centre Hospitalier Universitaire Vaudois (CHUV) and University of Lausanne, CH-1011 Lausanne, Switzerland

## Abstract

The splenium of the corpus callosum connects the posterior cortices with fibers varying in size from thin late-myelinating axons in the anterior part, predominantly connecting parietal and temporal areas, to thick early-myelinating fibers in the posterior part, linking primary and secondary visual areas. In the adult human brain, the function of the splenium in a given area is defined by the specialization of the area and implemented via excitation and/or suppression of the contralateral homotopic and heterotopic areas at the same or different level of visual hierarchy. These mechanisms are facilitated by interhemispheric synchronization of oscillatory activity, also supported by the splenium. In postnatal ontogenesis, structural MRI reveals a protracted formation of the splenium during the first two decades of human life. In doing so, the slow myelination of the splenium correlates with the formation of interhemispheric excitatory influences in the extrastriate areas and the EEG synchronization, while the gradual increase of inhibitory effects in the striate cortex is linked to the local inhibitory circuitry. Reshaping interactions between interhemispherically distributed networks under various perceptual contexts allows sparsification of responses to superfluous information from the visual environment, leading to a reduction of metabolic and structural redundancy in a child's brain.

## 1. Introduction

**Figure 1:**
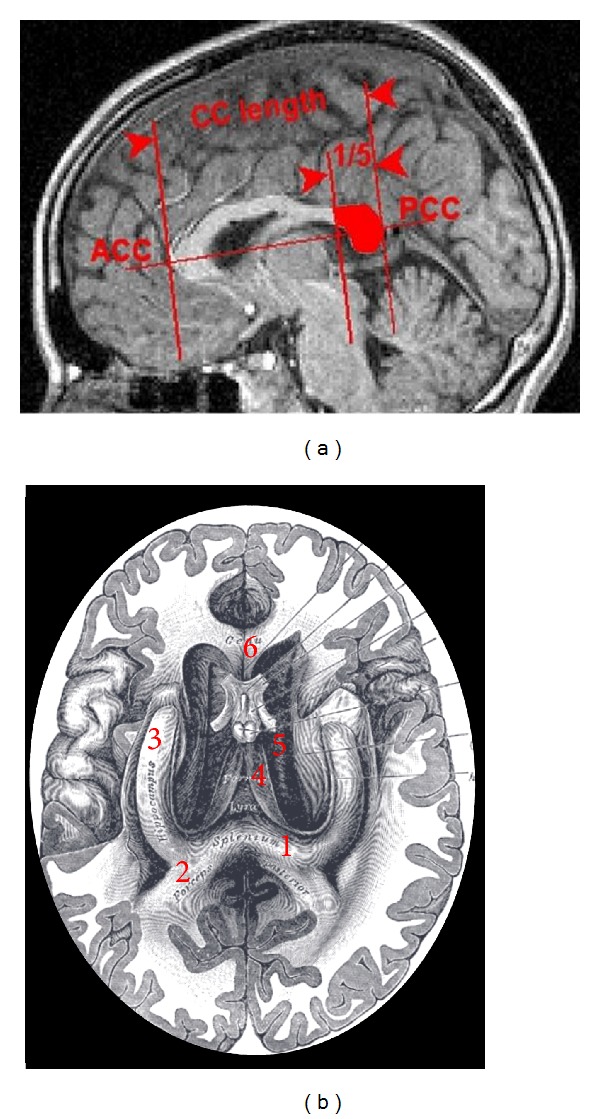
Midsagittal and axial views of the splenium. (a) Midsagittal T1-weighted MRI shows the corpus callosum (CC) and the splenium (in red). According to the conventional partitioning scheme, the splenium corresponds to the posterior 1/5 of the CC, which is separated by the border line perpendicular to the line linking the most anterior (ACC) and posterior (PCC) points of the CC. (b) Axial view of the splenium (1) from Gray's Anatomy of the Human Body. The numbers refer to the posterior forceps (2), hippocampus (3), fornix (4), undersurface of the CC (5), and genu of internal capsule (6).

The *splenium* is a name of the posterior part of the corpus callosum (CC). In Greek this word means a bandage strip tied around an injury or a damaged part of someone's body. Although the association of the name with the respective structure is not immediately clear from the most common sagittal images of the brain (Figure 1(a)), which create an illusion of the CC as a structure that can only be artificially partitioned, the basal view of the splenium from Gray's Atlas (Figure 1(b)) completely justifies its name and shows that the splenium fibers connect occipital and parietal cortices, as well as inferior and medial temporal regions (including the posterior cingulate).

According to anatomical tracing studies, the fiber composition of the splenium is heterogeneous: its anterior part includes thin late-myelinating fibers from parietal and medial temporal association areas, while the posterior part contains thick early-myelinating fibers linking primary/secondary visual areas [1–5]. Most of the splenium fibers are reciprocal and connect the hemispheres homotopically, while some fibers are heterotopic, although homoareal, and others link different cortical areas [6–9]. The splenium connections are unevenly distributed across cortical areas both in humans and in nonhuman primates [7, 10, 11]. They are relatively dense and widely distributed in the extrastriate cortices, whereas in the striate cortex, callosal fibers are located in a narrow strip along the V1/V2 border representing the vertical meridian of the visual field. 

These basic aspects of the splenium organization are supplemented by recent neuroimaging findings. *In vivo* tracing—diffusion tensor imaging (DTI)—studies describe a more detailed spatial organization of fibers within the human splenium [3, 5, 12]. According to these reports, the middle part of the splenium carries fibers connecting dorsal visual and association parietal areas, the superior-posterior part contains fibers linking posterior cingulate and retrosplenial cortices, and the inferior-anterior portion incorporates fibers from ventral visual areas. The neuroimaging data also question some features of splenial connectivity that had been established in animal and postmortem human studies. These include the notion that the primary visual cortex is mostly devoid of callosal connections: significant interindividual variability of connections between the striate cortices (with one-third of participants exhibiting direct interhemispheric projections in this area) has been found by Putnam and colleagues [12]. Another example is the assumed symmetry of callosal connections: greater interhemispheric connectivity from the right hemisphere to the left one has been found in the extrastriate cortices [12]. 

The diverse structural properties of the splenial fibers across brain areas suggest that they are involved in a variety of functions, while their considerable variation between subjects implies a contribution of the splenium to plastic changes in the course of human development. Considering that the splenium is well defined anatomically and is easily accessible in animal models and in noninvasive human neuroimaging, this structure is of significant interest for basic neuroscience and clinical applications. This paper addresses the structural and functional development of the splenium based on the recent literature with an emphasis on the heterogeneity of its functions and mechanisms at different levels of the visual hierarchy. 

## 2. Structural Development of the Splenium

The development of the human CC was studied using both *postmortem* and *in vivo* MRI-based techniques. A direct comparison of these methods in [13] showed that, at least in terms of the CC area and shape, they provide consistent information. Moreover, the two methods are complementary: while *postmortem* material allows a more precise identification of the CC borders, the *in vivo* imaging techniques are easily compatible with (neuro)psychological characteristics and permit a longitudinal study design, thus providing an inestimable advantage for the research into human development. The results of both approaches are discussed hereafter.

The developing splenial fibers travel together with the hippocampal commissure, whereas the frontal fibers of the CC cross the midline separately from the anterior and hippocampal commissures [14, 15]. Accordingly, the CC forms as a fusion of two separate segments. This developmental pattern as well as the partial CC ageneses and regional malformations suggests that the splenium can be considered a neocortical component of the hippocampal commissure [15], which carries fibers connecting the hippocampi together with those linking the posterior parietal, medial temporal, and medial occipital cortices of the two hemispheres [4, 5, 16].

Anatomical reports show that the prenatal development of the human CC is characterized by a posterior-to-anterior gradient, with the prominent splenium emerging only in the 18th or 19th week of gestation [14, 17–19]. After birth, the slower growth of the splenium compared to the genu is replaced by the opposite trend, with higher growth rates of the splenium than those of the genu [18, 20, 21]. Similar nonuniform postnatal growth of the CC compartments was demonstrated with MRI in baboons [22]. In particular, by postnatal week 32, their midsagittal splenium area achieves 55% of the average adult size, whereas the genu and the anterior midbody attain only about 50%. 

As can be extrapolated from the monkey data, the total number of callosal fibers continues to increase until birth [1]. Nevertheless, at the end of gestation and during the first months after birth, the sagittal area of the CC reduces both in monkeys [1] and in humans [13, 18]. Since this coincides with the time of massive axonal elimination, the latter is suggested to be the main cause of CC reduction [1, 13]. Further postnatal changes in the callosal sagittal area are interpreted as an interplay between continuing myelination, pruning, and the redirection of fibers [23]. 

Structural MRI-based studies report the prolonged growth of the total CC area and splenium (among other CC subdivisions) from birth adulthood in nonhuman primates, including chimpanzees [24], Bonnet macaques [25], and capuchin monkeys [26]. Since the end of the 1990s, several laboratories have applied mesh-based computational MRI techniques to the analysis of the sagittal callosal area in children and adolescents [27–30]. In this method, aimed toward longitudinal research, four-dimensional quantitative maps of growth patterns are reconstructed by computing a three-dimensional elastic deformation field, which rearranges the shape of the CC in the earlier scan into the shape in the later scan [30]. These groups reported greater increase in the splenium than in the anterior CC regions in children and adolescents aged 4–18 years [27, 28], 6–15 years [29], and 7–22 years [30].

Alternative imaging methods provide converging results. To assess the CC development in healthy children of 3–15 years, Kim and collaborators [31] used multiecho T2 relaxometry based on the longer T2 relaxation times of water molecules within the axon and extracellular space unbound to macromolecules. During development, the axonal diameters in the splenium grow in parallel with the reduction of their density [1, 2]. Therefore, the continuing increase of axonal size should correlate with the increase of T2 relaxation times. The measurements in genu and splenium revealed that the relaxation times significantly correlate with age only in the splenium, suggesting its prominent growth in the late childhood and adolescence. DTI studies, although inconsistent about the anterior-to-posterior gradient of CC maturation, nevertheless show that the splenium develops gradually through adolescence [32, 33].

Recently, in a large computational mesh-modeling MRI study of 190 children and adolescents aged 5–18 years, Luders and coauthors [34] confirmed that the callosal area increases with age and revealed the age-, sex-, and region-specific rates of growth. In particular, in a result qualitatively similar to previous neuroimaging studies (e.g., [29]), the younger children showed the most pronounced growth in the anterior CC, while the splenium began to overtake the anterior parts of the CC starting from the age of 9-10 years in girls and of 11-12 years in boys. 

A synthesis of the *postmortem* anatomical and *in vivo* MRI data suggests that periods of accelerated growth of the genu alternate with periods when the splenium picks up speed. Such shifts occur around birth time (the splenium speeds up compared to the genu), in early childhood (the genu begins to outrun the splenium), and in middle childhood (the splenium once more takes the lead in growth). The mechanisms behind these changes seem to be age-specific. In the context of the first postnatal spurt of splenium growth, the data of Chalupa and colleagues from their tract tracing studies in rhesus monkeys are of interest [35, 36]. They showed that, in late fetal development, the elimination of CC axons in the visual areas is less pronounced than that in the sensorimotor cortex. If the lower proportion of axonal retraction in the posterior areas is also characteristic for humans, this phenomenon could explain the higher splenium growth in the early postnatal period. 

The last period, characterized by an anterior-to-posterior gradient of the CC development, is in humans likely related to the protracted myelination of the splenium. Myelination starts at 3-4 months after birth and continues into adulthood [21, 37]. In adults only 16% of the CC fibers remain unmyelinated [2]. To analyze the link between CC area and myelination, Fornari and colleagues (2007) used magnetization transfer imaging (MTI) in children of 7 to 13 years of age [38]. MTI estimates the efficiency of magnetization exchange in biological tissues between a pool of free protons in intra- and extracellular water and a pool of protons bound to macromolecules (for review, see [39]). As shown in an *in vitro* experiment, the contribution of the myelin sheets to the MT contrast is nine times larger than the contributions of intra/extracellular water [40]. A postmortem study of the multiple sclerosis brain demonstrated highly significant correlations between morphometric and MTI measures of myelin content [41]. Since the most important contributors to the magnetization transfer effect are the extent, concentration, and integrity of myelin membranes, MTI permits an accurate evaluation of changes in myelination in children, aging people, and populations with myelination abnormalities [42–44]. Consistently with previously reviewed reports, in a group of healthy children, the most robust direct correlation between the MTI index of myelination and a child's age has been shown by Fornari and colleagues for the area of the splenium [38]. 

Myelination in the nervous system is a plasticity-dependent process [45]. The size of the CC in animals and humans increases with learning or training [46–48]. It is likely that nonmonotonic growth of the splenium probably reflects its plastic tuning to the heterochronically maturing visual functions in childhood and adolescence. More specifically, the accelerated growth of the splenium in the first postnatal weeks/months coincides with the fast development of sensitivity to orientation, direction of motion, and disparity [49]. Another period of relatively high growth rates that starts in middle childhood accompanies improvement of the functions associated with spatial integration (see Sections 5 and 6). 

## 3. Known and Assumed Mechanisms and Functions of the Splenium 

Before proceeding any further, it should be noted that the tasks performed by the CC within the framework of interhemispheric integration as well as the physiological mechanisms implementing these tasks remain to be studied further. At a functional level, basic physiological effects of the CC are conceptualized as excitation and inhibition. Specifically, excitation refers to the tendency of one site to activate the symmetric location in the other hemisphere, while inhibition refers to the opposite effect [50]. Since cortico-cortical long-distance connections are mainly excitatory, the interhemispherically induced suppression of a response necessarily includes local inhibitory interneurons. Therefore, the interhemispheric effects resulting from a summation of multiple diversified events at a neuronal/synaptic level require a very cautious interpretation at a network level, especially in noninvasive human research.

In a decades' long debate about the excitatory, inhibitory, or mixed nature of interhemispheric effects of the CC, the excitatory function seemed to get the majority of support. To this end, in 2005 Bloom and Hynd [50] wrote the following: “The available research, no matter how limited, primarily supports the notion that the corpus callosum serves a predominantly excitatory function.” Recent research has revealed a more complicated picture, in which the CC functions and mechanisms not only change along its anterior-to-posterior axis depending on the cortical area of origin/destination, but also vary within a singular area.

The application of sophisticated experimental methods by the group of Innocenti substantially enriched our understanding of the repertoire of splenial functions [51–53]. By combining local reversible thermal inactivation in one hemisphere with optical imaging of intrinsic signals or electrophysiological recordings in the other hemisphere, these authors showed that the splenium fibers connecting visual areas 17/18 of the ferret modulate the driving thalamocortical input by means of inhibitory effects at short latencies and of excitatory effects at longer latencies [52]. The latencies of inhibitory effects are compatible with higher conduction velocities of thick early-myelinating fibers, whereas the excitation is apparently driven by thinner axons with lower conduction velocities. All the modulatory influences are stimulus-specific [53]. Their interplay with axonal geometry can change the synchronization of stimulus-driven local field potential [51]. Considering that synchronization serves to recruit neuronal populations to common activity [54, 55], such effects of the splenium might not be limited to the area of their destination—a narrow strip at the 17/18 border—but affect the functionality of a significant part of the area (see Section 4). 

Not much is known about the splenium functions in the extrastriate areas. However, comparing the splenium connections between the striate cortex, where they are thick (heavily myelinated), sparse, and concentrated along the border, and extrastriate cortices, where interhemispheric connections are thin, dense, and widely distributed [7], it is difficult to escape the conclusion that the functions of splenium fibers vary across visual areas. The conventional assumption is that the functional role of the splenium in a particular extrastriate area is defined by its specialization. For instance, Olavarria and Abel (1996) [56] reported that callosal cells are assembled in regular protrusions into V2 of the monkey. These protrusions are distributed along the V1/V2 border at the intervals corresponding to the arrangement of thick and thin stripes. Given that the stripes are specific to the organization of the V2 and correspond to the functional streams engaged in the processing of orientation and direction [57, 58], this structural evidence suggests some area-specific functions of the splenium beside establishing continuity across the visual field. 

One such function is figure-ground segregation, which refers to the ability of the visual system to segment images of the external world into objects and background. To this end, a mechanism has been proposed for the isolation of a figure from the background through the detection of its borders [59, 60]. It relies on inhibition among neurons with neighboring receptive fields tuned to the same feature. As a result, within a homogenous region, similarly tuned neurons mutually inhibit their activity, whereas at borders, such neurons are less inhibited due to regional heterogeneity. The receptive fields that implement this border-detecting mechanism are characterized by center-surround antagonism, that is, they have a receptive field center that is excited by a particular image feature and surround that is inhibited by the same feature. Desimone and colleagues (1993) found that, in V4 of the monkey, the classical receptive fields (excitatory centers) are mostly limited to the contralateral visual field, while their suppressive surround might extend into the ipsilateral visual field up to 16° from the vertical meridian [61]. In these experiments, dissection of the CC abolished much of the inhibition from the ipsilateral part of the surround, demonstrating its involvement in the core mechanisms of figure-ground segregation implemented in the V4. 

Callosal connections are structurally, functionally, and developmentally similar to long-range intrahemispheric corticocortical connections [11, 62]. With the exception of the CC agenesis, there are no pathologies in which they are specifically involved [63]. Nonetheless, since intrahemispheric mechanisms within a single level of the visual hierarchy are realized via lateral intracortical horizontal fibers and short-range association fibers (U-fibers), the number of which is orders of magnitude greater than that of splenial fibers executing the same functions interhemispherically [64], one may speculate that the CC should have some adaptations compensating for its limited number of connections, and, therefore, interhemispheric networks should differ from the respective intrahemispheric networks. 

Finally, the functions of the splenium may encompass communication among different levels of hierarchy. The inactivation of higher-order visual areas weakens the suppressive surround of neurons in lower-order areas, suggesting a role for top-down connections in this mechanism [65]. The heterotopic splenial fibers [6, 9], especially those between association and primary visual areas, could mediate such feedback. 

## 4. Development of Interhemispheric Synchronization in the Visual Brain

As stated in the previous section, the interhemispheric synchronization of network activity can be involved in a variety of functions. The impact of the splenium in synchronizing the electrical activity between the hemispheres is supported by animal models and noninvasive human studies [66–68]. Kiper and colleagues [67] examined interhemispheric synchronization in ferrets, in which, like in other mammals, the splenium fibers located along the V1/V2 border selectively connect neurons with the receptive fields having similar orientation preferences and placed near the vertical meridian of the visual field. For this structure of connectivity, the binding-through-synchronization hypothesis [55] predicts an increase of interhemispheric synchronization in response to the bilateral collinear stimuli near the vertical meridian compared to the noncollinear stimuli.

Indeed, by contrasting differently oriented and located bilateral gratings before and after the section of the CC, the authors have shown that interhemispheric synchronization of epidural EEG increases in response to the isooriented gratings near the vertical meridian compared to the orthogonally oriented gratings, whereas callosotomy abolishes the effects of stimulus configuration. The same set of stimuli used in a noninvasive human study [68] induces similar changes of interhemispheric synchronization in surface EEG, whereas the reduction of interhemispheric synchronization in the absence of the splenium in humans was shown in acallosal and split-brain individuals [69–71].

It is safe to assume that even less dramatic changes in interhemispheric connectivity that occur in human postnatal development, for example, myelination of the splenium fibers, would also affect the interhemispheric synchronization of neural networks. The network activity of the brain is oscillatory in nature. Oscillations provide a temporal frame for neuronal firing by means of synchronization of pre- and postsynaptic potentials [54, 55]. In the context of this discussion, oscillations in the EEG alpha band are of special interest. First, the alpha rhythm is the most prominent oscillatory activity that can easily be recorded by means of noninvasive surface EEG within a wide range of ages. Second, it is generated by *visual* cortical circuits interacting with thalamocortical loops [72, 73] and has a relatively narrow frequency range between 8 and 12 Hz. Third, the alpha rhythm is characterized by a protracted course of development in children [74, 75] comparable with that of the CC.

In 7- to 12-month-old infants, the activity that can be recorded over the occipital-parietal cortex within the frequency range 5–9 Hz has the properties of alpha rhythm and is considered its precursor [76]. Alpha peak frequency logarithmically increases with age [75], providing the best estimate of maturation among the EEG parameters [74, 77]. In parallel, the spatial organization of alpha rhythm develops. In a high-density EEG study, Srinivasan showed that, at the peak alpha frequency, the 6- to 11-year-old children demonstrate lower long-range synchronization between the anterior and posterior Laplacian EEG signals in comparison to the young adults [78]. Thus, the typical feature of adult EEG—high coherence between distant EEG signals in the alpha band—is still absent in middle childhood. 

Farber and Knyazeva demonstrated an immaturity of long-range interactions for the case of interhemispheric connections [79]. They analyzed the development of the interhemispheric coherence of alpha rhythm in 320 healthy children and adolescents aged 2–17 years. Interhemispheric synchronization rapidly increased with age in early childhood (between 2 and 7 years), whereas in middle childhood and adolescence the increase rate progressively slowed down. This developmental trajectory was also best approximated by logarithmic function. The striking similarity between the trajectories of the alpha frequency and synchronization development and that of the white matter maturation [80, 81] suggest that the processes are closely related.

Theoretically, the frequency of coupled oscillators depends on connection strength and time delays between them [82, 83]. To this end, combined EEG-DTI studies have found that, in adults, individual alpha frequency is linked to the structural properties of corticocortical and thalamocortical connections [84, 85]. The strongest correlation between an individual alpha frequency and fractional anisotropy, which reflects the joint contribution of fiber density and myelination, was found for the splenium. 

To summarize, although studies directly analyzing links between interhemispheric alpha synchronization and structural maturation of the splenium remain to be performed, the development of alpha rhythm in children seems to be closely linked to the maturation of the CC. Moreover, the increase of interhemispheric alpha synchronization with age implies that the long-range interhemispheric interactions become an increasingly important regulator of visual functions. On the other hand, the relatively low level of functional cooperation between the hemispheres in the immature brain suggests the predominance of local intrahemispheric mechanisms underlying vision in young children. 

## 5. Visual Functions with a Protracted Course of Development 

The extended structural and functional maturation of the splenium inspires me to consider the perceptual functions with protracted developmental trajectories, although it is not clear *a priori* whether such a gradual development depends on the inter- or the intrahemispheric mechanisms. Most visual functions achieve adult levels within the first few months (e.g., contrast, motion, and orientation sensitivity) or the first few years (grating acuity and binocularity) of postnatal life. In contrast, visual spatial integration (SI) develops slowly. SI refers to the processes that assemble local information across the visual field to implement a global representation of spatially extended objects in the brain. Behavioral experiments consistently show that the basic mechanisms of spatio-temporal integration are available in the first months or even weeks of human life. Infants treat the coherently moving parts of a display as belonging to the same object [86], differentiate upright from inverted biomotion displays [87], and integrate component motions into coherent pattern motion over large regions of space [88]. 

Yet the development of perceptual organization abilities takes a long trajectory through childhood and adolescence. Thus, sensitivity to global form in glass patterns is adult-like only at 9 years of age [89]. In a contour-detection task, children significantly improve grouping operations between 5 and 14 years of age [90]. Sensitivity to biological and global motion advances between 6 and 14 years of age [91]. Experiments with complex visual displays like hierarchical shapes and compound letters reveal that even in adolescence visual perception is biased toward representing local elements [92, 93]. Furthermore, the organization principles, working in early life, improve with age and so does the ability to use collinearity for the integration of spatially distant line segments, which increases at least until 10 years of age [94]. The neural basis of this protracted course of functional maturation is discussed in the following sections. 

## 6. Myelination of the Splenium Shapes Functional Activation in Extrastriate Areas

In adults, cognitive performance correlates with the size of the callosal area [95] and cognitive impairment with the demyelination of the splenium [96]. Apparently, myelination facilitates interhemispheric interaction by enhancing the coordination of interhemispheric input [97], which leads to a more efficient recruitment of the target neural population to common activity [98, 99]. If this is the case for the developing splenium in children, a correlation between its myelination and the activation of respective networks is to be expected.

**Figure 2:**
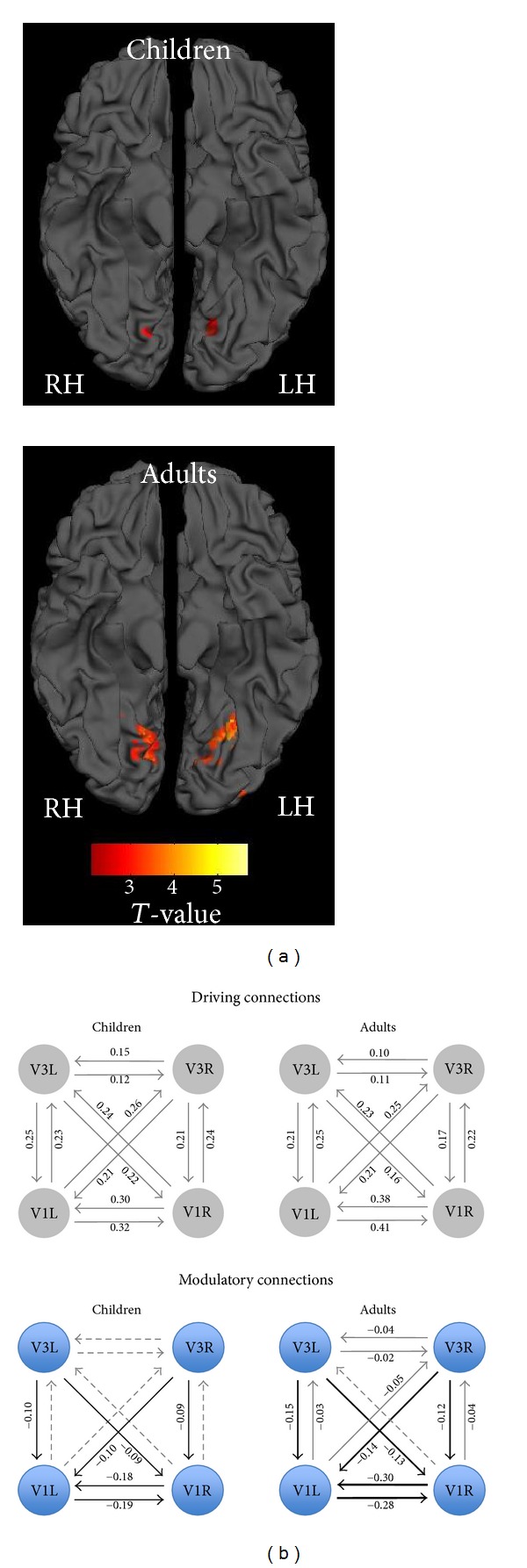
Interhemispheric integration effects as revealed by fMRI activation and dynamic causal modeling. (a) Statistical maps of the CG > NG contrast for the children and adults groups superimposed on a pial surface of a standard brain in MNI space (bottom view). In both groups, an increase of BOLD response is located within the lingual/fusiform gyri, but in the adults it is higher and more extensive. The center of each cluster served to define the V3 location for DCM analysis in each group. (b) Intrinsic and modulatory connections in children and adults. Gray/blue-filled circles symbolize the brain regions involved in the modeled network. They are located in the left and right primary visual cortex (V1L and V1R, resp.) and in the left and right V3v (V3L and V3R, resp.). Arrows between the circles stand for the bidirectional intrinsic/modulatory connections. Dashed arrows designate nonsignificant connections; gray arrows, significant but not changing with age; black arrows, significant and changing with age. The average estimate of the strength of connection in Hertz is shown alongside the respective arrow.

To test this in [38], we used a simple interhemispheric paradigm that requires only passive viewing of visual stimuli, verified earlier by us [67, 68] and by others in animal and human experiments. Being minimally demanding, this task is applicable to groups of various age and health across the lifespan. Specifically, subjects fixated on large high-contrast bilateral gratings including horizontal collinear coherently drifting gratings (stimulus CG) and noncollinear orthogonally oriented and drifting gratings (stimulus NG). Of the two stimuli, only CG is fusible into a single image, while the NG is expected to induce a segmentation of the image between the right and left visual fields. Functional magnetic resonance imaging (fMRI) shows that, across different age groups, the contrast CG > NG manifests highly reproducible activations (Figure 2(a)) in the ventral-stream V3v/V4 areas [38, 98–100]. In adults, these activations correlate with interhemispheric EEG synchronization [98, 99] and, therefore, can be considered a neural substrate of interhemispheric integration. 

First, we investigated whether the activation of these integration-specific areas is affected by splenium maturation. Children of 7–13 years of age were scanned while they viewed the gratings [38]. By implementing fMRI and MTI protocols in the same scanning session, we could estimate both functional and structural aspects of interhemispheric interaction. Each stimulus induced widespread activation over the striate and extrastriate areas. The activation associated with the CG > NG contrast was limited in children to the V3v part of the adults' activation (Figure 2(a)). This modulation of BOLD signal manifested by the networks presumably involved in the interhemispheric integration was correlated with the myelination of the splenial system of fibers [38]. Apparently, by changing the speed of transmission and the effective geometry of the CC fibers, myelination allows well-synchronized input to the opposite hemisphere, resulting in enhanced activation [97–99]. This effect points to the excitatory aspect of splenium function.

## 7. Transsplenial Inhibition in Adults and Children

In order to test other aspects of the development of interhemispheric interaction via the posterior callosal connections, we reanalyzed the fMRI time series from this experiment [100] with dynamic causal modeling (DCM), a method for evaluating effective connectivity, that is, the influence that one local neural system (source) exerts on another (target) [101, 102]. DCM differentiates positive coupling (excitation) that results in correlated increased activity between source and target regions from negative coupling (inhibition) that leads to a relative decrease in the target activation compared to the source. Although the term *inhibition* is conventionally used in the DCM literature, its true meaning in this context is the *suppression* of activation response due to a variety of processes at a cellular level, including inhibition per se. 

The visual interhemispheric integration task described in the previous section is wellsuited for modeling effective connectivity since its neural substrate is a relatively restricted network, the nodes of which can be clearly identified, and the effects of the stimuli can be described in terms of factorial design. The latter allows one to model main factors as driving context-independent effects (in this case, stimulation with any grating stimulus compared to gray-screen (background)) and interactions, resulting from experimental manipulations, as modulatory (context-dependent) effects (here it is the effect of interhemispheric integration in response to CG compared to any grating stimulus). Specifically, DCM allows an analysis of such an interaction in terms of modulatory connections, that is, by defining their architecture and the character of effect. 

We used two pairs of interhemispherically symmetric regions for the model: one pair in the primary visual cortex, where the driving input arrived, and another pair in the extrastriate visual cortex, where the response varied depending on the stimulus (Figure 2(b)). The nodes were limited to the 4 mm radius spheres centered on the local maxima within these predefined territories. According to the probabilistic cytoarchitectonic atlas [103], one pair of nodes in each hemisphere occupied the territory on both sides of the V1/V2 border, while another one was located at the V3v/V4 border (Figure 2(b)). In this model, the driving signals induced by visual stimulation arrive in the left and right primary visual cortices (V1L and V1R nodes of the model) and spread within the model between the V1L, V1R, V3L, and V3R nodes by means of reciprocal intrahemispheric, interhemispheric, homotopic, and heterotopic connections. On the assumption that each of these intrinsic connections can be modulated, the structure of modulatory connections reproduced the architecture of intrinsic connections. We used this model for comparison of children (the same group of 7–13 years as in [38]) and adults that viewed the same gratings.

The intrinsic (driving) effective connections (all excitatory) between the visual areas were significant in both groups and did not differ between children and adults, in keeping with a large body of evidence that basic visual networks integrated via long-distance reciprocal pathways are established early in the course of development. The modulation induced by the CG stimulus was conveyed by lateral and feedback connections, all of which were inhibitory. The strongest modulation manifested by strengthened mutual suppression was found between the primary visual areas in both subjects' groups.

A recent noninvasive human study provided converging evidence of transsplenial inhibition of neural responses [104]. In these experiments of Bocci and colleagues, the splenium input was manipulated with transcranial magnetic stimulation (TMS), the effects of which were assessed with visual evoked potentials (VEPs) in response to the whole-field horizontal gratings. Similar to the bilateral collinear gratings (stimulus CG) used by Fornari with colleagues [38] and Knyazeva with colleagues [98, 99], this stimulus was interhemispherically identical. The unilateral TMS of V1 increased the amplitudes of VEP components generated in the striate and extrastriate areas of the contralateral hemisphere in response to the stimuli of medium-to-high contrast. Considering that TMS imposes inhibitory effect, that is, excludes callosal input, the increase of VEP can be attributed to disinhibition.

Both our DCM results and the reviewed human findings are remarkably similar to the evidence from the already cited experimental study [52], in which the local cooling of area 17/18 in one hemisphere of the ferret reversibly eliminated callosal input to the symmetric area in the intact hemisphere. The effect of this manipulation consisted largely in the decrease of local field potential (LFP) in response to whole-field orthogonally oriented gratings and in the increase of LFP to isooriented gratings. 

A plausible interpretation encompassing all these findings is that orthogonally oriented gratings essentially represent two different stimuli, which activate the networks with different orientation and/or direction preferences through the thalamocortical and callosal pathways, while isooriented gratings activate the neurons similarly tuned in both hemispheres, thus extending their network over the two hemispheres. As a result, the orthogonally oriented gratings induce segmentation, while collinear gratings bring on integration between the visual hemifields. The basis of integration for large high-contrast gratings at the V1 level is “no change in stimulus properties,” that is, no borders. Such stimuli are known to induce especially strong surround suppression, leading to a sparse population response [105–107]. If this account holds true, the net result of converging thalamocortical and callosal inputs induced by a strong visual stimulus extending into both hemifields would be a suppression of the V1 response. 

Therefore, the splenium can be involved in the adaptive process of neuronal response sparsification through suppressive mechanisms activated by redundant visual information. In a natural vision, when the entire retina is simultaneously stimulated, such a mechanism is essential for the efficient processing of moving images [105, 107]. Moreover, it is likely that inhibition is more important for the processing of visual information in an awake animal than anesthetized animal models suggest [97, 108].

In addition to the lateral effective connections between the primary visual areas, effective feedback connections from the extrastriate V3v/V4 nodes convey inhibitory modulation induced by the isooriented stimulus in both groups (Figure 2(b)). This is consistent with animal models, where the large spatial extent of surround suppression together with its short latent period suggests the involvement of feedback signals from the extrastriate cortex transmitted by fast myelinated fibers [65, 109, 110].

In our DCM model, the inhibitory feedback is carried by heterotopic interhemispheric connections. Since there are no assumptions about the number of synapses implementing a connection in DCM, it remains to be demonstrated whether the heterotopic callosal connections shown in animals and humans [6, 9] are implicated. The experiments of Ban and colleagues (2006) suggest such a possibility [111]. They have found that the BOLD response to the arcs presented symmetrically in the lower visual field quadrants is significantly lower compared to the response to the same arcs located asymmetrically (diagonally). In the absence of direct interhemispheric V1 connections between the low and high visual quadrants, this change of V1 activation is likely due to the top-down influences from the extrastriate areas. The shortest pathway for such an effect would be the heterotopic splenial fibers [9]. 

## 8. Formation of Interhemispheric Inhibition with Age: Some Implications for Development

As demonstrated by Lassonde and colleagues, children younger than 10 years of age show remarkably small deficits after callosotomy [112, 113]. Although visual functions largely escaped examination, the set of various tasks including intermanual comparisons and naming of shapes and objects, as well as localization of touch, leave few doubts about close-to-normal performance even at their first neuropsychological assessment after the surgery and about the remarkably fast compensation of residual deficits. In contrast, children older than 10 years of age and adolescents show a full-blown split-brain syndrome. Similar to adult split-brain patients, these children demonstrate a breakdown in interhemispheric communication, including the loss of intermanual transfer and integration of tactile information and difficulty naming objects held in the nondominant hand. Nevertheless, they recover more rapidly and completely than adults [112].

Cumulatively, the data of Lassonde and colleagues suggest that some functions of the immature CC can be shared with alternative pathways, thus accounting for minimal postoperative deficits in young children. However, continuing development leads to the cortex rewiring through elimination of overproduced connections [23]. The resulting patterns of connectivity may have a limited capacity for reorganization. Ptito and Lepore obtained direct evidence in favor of this view in experiments on cats with the posterior CC sectioned either before this structure reached maturity or after its maturation [114]. To disconnect each eye from the contralateral hemisphere, all these animals had the optic chiasma sectioned in adulthood and then were monocularly trained on a visual discrimination task. Only cats with early callosal transsection showed a capacity for the interhemispheric transfer of pattern discriminations. Thus, in parallel with CC maturation, other connections become inaccessible, limiting plastic postoperative changes with age.

Yet the majority of functions are probably not strongly reorganized in the ontogenesis but gradually improve with CC development. Our DCM-based findings shed new light on the nature of callosal functions with a protracted course of development [100]. Specifically, in contrast to excitatory connections that show no signs of changes between children and adults, interhemispheric modulatory connections (both lateral and descending) strengthen with age (Figure 2(b)). The increase of interhemispheric suppression in the primary visual cortex of adults compared to that in children was the strongest effect observed. Interestingly, although the strength of inhibitory connections correlated with age, it did not correlate with the MTI indices of splenium myelination [100]. This is in line with previously reviewed experimental evidence for the involvement of fast, that is, thick and early-myelinating, fibers in interhemispheric inhibitory effects [52].

Alternatively, since the CC neurons are generally excitatory but may target local inhibitory neurons [7], interhemispheric inhibition can be implemented via polysynaptic pathways with long-distance excitatory and local inhibitory components. Then the correlation with age in the absence of a correlation with myelination apparently reflects the development of local connections. Indeed, the local GABAergic mechanisms of the primary visual cortex analyzed postmortem manifest the extended development, which continues well into the second and third decades of life [115]. 

It should be noted that from an ontogenetic perspective, the prolonged formation of transsplenial modulation between the primary visual areas challenges the conventional view that posits the prior maturation of the early visual cortex as a precondition for the later development of higher-order ventral stream regions [116].

The modulatory effects transmitted in our model via interhemispheric top-down effective connections are also weaker in children than those in adults. Considering the ages of the children in this group (7–13 years), the DCM evidence points to the slow formation of feedback connections, which might be a part of the neural network that enables collinearity detection [90]. The available data on their structural maturation are limited to the connections between V2 and V1 [117, 118]. According to these postmortem anatomical studies, the upper layers of V1, which receive the feedback and callosal connections, seem to be immature at 5 years of age. 

The reviewed literature together with structural and functional MRI, EEG, and DCM evidence obtained by the author's group points to a slow structural development of the splenium in human ontogenesis and to a gradual formation of transsplenial effective connections conveying inhibitory influences. An important outcome of the protracted maturation of the mechanisms with splenial involvement is a greater efficiency of neuronal networks. Reshaping interactions between interhemispherically distributed networks under various perceptual contexts allows sparse responses to superfluous information from the visual environment. Another aspect of these processes is a reduction of well-known metabolic and structural redundancy in children's brains [23, 119].
